# A Multi-Center Study on the Relationship Between Developmental Regression and Disease Severity in Children With Autism Spectrum Disorders

**DOI:** 10.3389/fpsyt.2022.796554

**Published:** 2022-03-09

**Authors:** Chaoqun Hu, Fan Yang, Ting Yang, Jie Chen, Ying Dai, Feiyong Jia, Lijie Wu, Yan Hao, Ling Li, Jie Zhang, Xiaoyan Ke, Mingji Yi, Qi Hong, Jinjin Chen, Shuanfeng Fang, Yichao Wang, Qi Wang, Chunhua Jin, Tingyu Li, Li Chen

**Affiliations:** ^1^Growth, Development and Mental Health Center of Children and Adolescents, Chongqing Key Laboratory of Childhood Nutrition and Health, National Clinical Research Center for Child Health and Disorders, Ministry of Education Key Laboratory of Child Development and Disorders, Children’s Hospital of Chongqing Medical University, Chongqing, China; ^2^Department of Developmental and Behavioral Pediatric, The First Hospital of Jilin University, Changchun, China; ^3^Department of Children’s and Adolescent Health, Public Health College of Harbin Medical University, Harbin, China; ^4^Department of Pediatrics, Tongji Hospital, Tongji Medical College, Huazhong University of Science and Technology, Wuhan, China; ^5^Department of Children Rehabilitation, Hainan Women and Children’s Medical Center, Haikou, China; ^6^Xi’an Children’s Hospital, Xi’an, China; ^7^Child Mental Health Research Center of Nanjing Brain Hospital, Nanjing, China; ^8^Department of Child Health Care, The Affiliated Hospital of Qingdao University, Qingdao, China; ^9^Maternal and Child Health Hospital of Baoan, Shenzhen, China; ^10^Department of Child Healthcare, Shanghai Children’s Hospital, Shanghai Jiao Tong University, Shanghai, China; ^11^Children’s Hospital Affiliated to Zhengzhou University, Zhengzhou, China; ^12^NHC Key Laboratory of Birth Defect for Research and Prevention, Hunan Provincial Maternal and Child Health Care Hospital, Changsha, China; ^13^Deyang Maternity & Child Healthcare Hospital, Deyang, China; ^14^Department of Children Health Care, Capital Institute of Pediatrics, Beijing, China

**Keywords:** autism spectrum disorder, regression, disease severity, relationship, multi-center study

## Abstract

**Introduction:**

This study aimed to investigate the prevalence of developmental regression in children with Autism Spectrum Disorder (ASD) and to explore its relationship with disease severity.

**Methods:**

We finally included 1,027 ASD children aged 2–5 years from 13 cities in China: 138 with regressive ASD and 889 with non-regressive ASD. The Social Responsiveness Scale (SRS), Autism Behavior Checklist (ABC), Child Autism Rating Scale (CARS), and Children Neuropsychological and Behavioral Scale-Revision 2016 (CNBS-R2016) were used to evaluate the core symptoms and developmental status of children in the two groups.

**Results:**

Among the 1,027 ASD children eventually included, 138 (13.44%) cases showed regressive behavior and the average regression occurring age was 24.00 (18.00–27.00) months. Among the regressive children, 105 cases (76.09%) had language regression, 79 cases (57.25%) had social regression, and 4 cases (2.90%) had motor regression. The total scores of ABC and the sub-score of sensory and stereotypic behavior (β = 5.122, 95% CI: 0.818, 9.426, *P* < 0.05; β = 1.104, 95% CI: 0.120, 2.089, *P* < 0.05; β = 1.388, 95% CI: 0.038, 2.737, *P* < 0.05), the SRS total scores and the sub-score of autistic mannerisms (β = 4.991, 95% CI: 0.494, 9.487, *P* < 0.05; β = 1.297, 95% CI: 0.140, 2.453, *P* < 0.05) of children in the regressive group were all higher than the non-regressive group. The total developmental quotient (DQ) of CNBS-R2016 and the DQ of gross motor, fine motor, adaptive behavior, language (β = −5.827, 95% CI: −11.529, −0.125, *P* < 0.05) and personal society in the regressive group were lower than the non-regressive group and the proportion of children with intelligent developmental impairment was higher the non-regressive group.

**Conclusion:**

Regressive autism is mainly manifested as language and social regression. Children with regressive ASD have more severe core symptoms, lower neurodevelopmental level DQ, and more serious disease degree than children with non-regressive ASD, which requires further etiological examinations and more clinical attention.

## Introduction

Autism Spectrum Disorder (ASD) is a complex neurodevelopmental disorder characterized by persistent impairments in social interaction and communication, and the presence of restrictive and repetitive patterns of behavior, interests and activities, and sensory anomalies (1). The latest report in the United States shows that the prevalence has risen from 1.7% in 2018 (2) to 2.27% in 2021 (3). In China, the prevalence was 0.7% in 2020 (4). The onset of behavioral signs of ASD is usually conceptualized as occurring in one of three ways: an early onset pattern, in which children demonstrate delays and deviances in social and communication development early in life; a regressive pattern, in which children develop largely as expected for some period and then experience a substantial decline in or loss of previously developed skills; and a developmental stagnation pattern, which is characterized by intact early skills that fail to progress or transform into more advanced developmental achievements (5). Developmental regression in ASD is typically defined as a child’s developmental milestones (language, social, motor, and other skills) at the corresponding age stage that have stabilized for more than 3 months, and then a single or multiple behavioral regression occurs. One commonly utilized criterion for language regression, complete loss of expressive language skills after acquiring at least five words and using them for at least 3 months (6). Previous findings indicate that most children with ASD showed an early onset pattern, but recent studies have shown that the frequency of regressive patterns is higher than before (7, 8). At present, there is still a lack of large-scale surveys on the relationship between the developmental regression of ASD children and the disease severity in China. Therefore, this study investigated the developmental regression of children with ASD in 13 cities across the country, and explored the relationship between the type of ASD and the severity of the disease, in order to provide a basis for clinical diagnosis and treatment.

## Materials and Methods

### Patients

All research participants aged 2–5 were recruited from the China Multi-center Preschool Autism Project (CMPAP). From May 2018 to December 2019, the project recruited ASD children from 13 cities where the project sub-centers are located-northern, eastern, western, southern, and central China. According to the completion of the questionnaire, it was confirmed that the effective sample size of 1,027 cases was included. The area where the sub-centers are located is defined as: the north (Heilongjiang, Qingdao, and Changchun); the east (Shanghai and Nanjing); the west (Chongqing, Deyang, and Xi’an); the south (Shenzhen, Hainan, and Hunan); the middle (Wuhan and Zhengzhou).

### Selection Criteria

Autism Spectrum Disorder children were recruited from outpatient clinics and special education institutions. The diagnosis of ASD was performed by psychiatrist, psychologists, or developmental behavioral pediatrician with extensive experience and using the Diagnosis and Statistical Manual of Mental Disorders-fifth edition (DSM-5) (9). ASD children with the following conditions were excluded: (1) children with brain injury; (2) children with severe physical and sensory impairment (blindness, deafness); (3) independent neurodevelopmental disorders and neurological diseases; and (4) other acute and chronic diseases. The inclusion and exclusion criteria of the regression group in this study were: (1) A detailed description of regressive behavior was the basis for inclusion. Parents needed to explain the detailed time point at which the behavioral skills previously mastered by ASD children began to regress, such as social, language, and motor. (2) “Regression” was defined as the loss of these abilities after appearing and remaining for more than 3 months, rather than being lost within a short period of time after the abilities appear. (3) Language regression was defined as loss of more than five spoken words used communicatively in children more than 18 months of age. For social and motor regression, when there was a clear indication of loss of social interest or motor skills, it was determined as regression regardless of age.

### Scales and Questionnaires

Autism Spectrum Disorder Child Health Questionnaire includes detailed content such as basic child information, family environment information, health status after birth, and description of developmental regression. Among them, the developmental regression includes: whether there is regression, the regression occurring age, and the detailed description of the regression behavior.

### The Autism Behavior Checklist

Autism Behavior Checklist (ABC) consists of 57 items in five dimensions, including sensory, relating, stereotypic behavior, language, and social self-help. Which is completed by parents to describe the behavioral characteristics of children with ASD. A score of 53 was used as thresh-old value for the diagnosis of autism, while scores for children with autism should be ≥67 (10).

### The Social Responsiveness Scale

A scale filled out by parents to assess the social ability of autism. A total of 65 items consists of 5 subscales including social awareness, social cognition, social communication, social motivation and autistic mannerisms. Normal children’s assessment score should be <65. The higher the total score, the more severe the social disorder (11).

### Child Autism Rating Scale

This scale is used by professionals to assess the symptoms and severity of autism, and is suitable for children over 2 years old. A total of 15 items are scored using four levels of 1, 2, 3, and 4. The total score is 30–36 points, and the items below three points are less than five items indicates mild-moderate autism; the total score is ≥36 points and at least 5 items with a score higher than three points indicates severe autism (12).

### The Revised Children Neuropsychological and Behavior Scale

It is assessed by professionals and used to assess the neurodevelopmental level of children aged 0–6 in the six domains of gross motor, fine motor, adaptive behavior, language, personal society, and autism warning behavior. DQ < 70 in each domain indicates developmental disorders. The warning behavior score <7 indicates normal, and >30 indicates highly suspected ASD, which can be used for risk identification and prediction of ASD (13).

### Statistical Analysis

SPSS statistical software 26.0 was used for statistical analysis. The Kolmogorov–Smirnov goodness-of-fit test was used to test the distribution of each dataset for normality before analysis. Continuous variables were described as mean ± standard deviation (M ± SD) and medians (inter-quartile ranges) [M (IQR)]. Categorical variables were described as *n* (%). Differences in demographic data betweeen groups were assessed by using the Chi-Square test or Mann–Whitney test. Multivariate (adjusted for age and gender) linear regression models were used to compare the scores of the Autism symptom scale and developmental level scale between the regressive and non-regressive groups. A *P* < 0.05 was considered statistically significant.

## Results

### Demographic Characteristics

**FIGURE 1:**
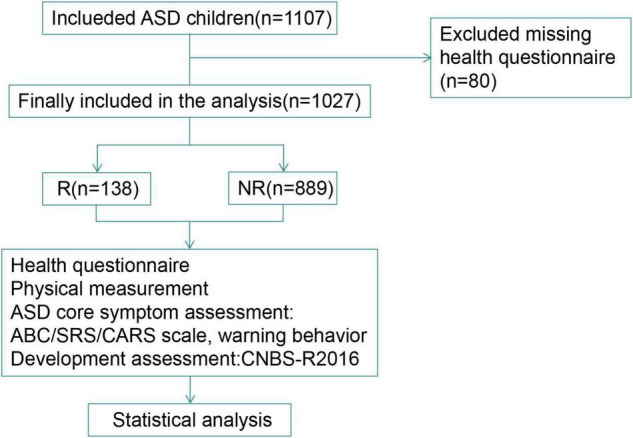
Flowchart of the study participants. R, regressive group; NR, non-regressive group.

According to the inclusion and exclusion criteria, a total of 1,027 ASD children aged 2–5years were enrolled in this study (Figure 1), with an average age of 3.55(2.95–4.18) years, including 844 males and 183 females (male: female = 4.61:1). There were 138 (13.44%) children had regressive autism (Regressive group, R) with a median (IQR) age of 3.64(2.90–4.24) years and 889 (86.56%) children had non-regressive autism (Non-regressive group, NR) with a median (IQR) age of 3.54(2.95–4.18) years. There was no significant difference in the age of children between the two groups (*z* = −0.329, *P* = 0.742) (Table 1). There was also no significant difference in region and residence (*x*^2^ = 4.211, 0.198, *P* = 0.378, 0.906).

**TABLE 1:** Demographic characteristics of the participants in R and NR groups.

Variable	R (*n* = 138)	NR (*n* = 889)	*Z*/x^2^	*P*
Average age (years), median (IQR)	3.64(2.90–4.24)	3.54(2.95–4.18)	*z* = −0.329	0.742
**Gender, *n* (%)**			*x*^2^ = 0.010	0.922
Male	113(81.88)	731(82.22)		
Female	25(18.12)	158(17.77)		
**Region, *n* (%)**			*x*^2^ = 4.211	0.378
North	37(26.81)	235(26.43)		
East	12(8.70)	112(12.60)		
West	44(31.88)	221(24.86)		
South	27(19.57)	194(21.82)		
Middle	18(13.04)	127(14.29)		
**Residence, *n* (%)**			*x*^2^ = 0.198	0.906
Urban	102(73.91)	671(75.48)		
Rural	34(24.64)	204(22.95)		
Miss	2(1.45)	14(1.57)		

*Data was shown as Median (IQR) or number (percentage). Chi-square test and Mann–Whitney test were used in the analysis. R, regressive group; NR, non-regressive group; IQR, interquartile range.*

### General Description of Developmental Regression

The average age of the onset of regression was 24.00(18.00–27.00) months. Among them, the regression rate of male children was 13.40% (113/843), and that of female children was 13.66% (25/183). The difference was not statistically significant (*x*^2^ = 0.010, *P* = 0.922). Among the regressive children, 105 cases (76.09%) had language regression, 79 cases (57.25%) had social regression, and 4 cases (2.90%) had motor regression (Table 2).

**TABLE 2:** Detail description of the regressive skills.

Variable	Frequency (*n* = 138)	Percentage
Language regression	58	42.03
Social regression	33	23.91
Language and social regression	43	31.16
Language and motor regression	1	0.72
All three of the above	3	2.17

### Comparison of Autism Behavior Checklist, Social Responsiveness Scale, Child Autism Rating Scale, and Communication Warning Behavior Scores Between Regressive Group and Non-regressive Group

The results of multivariate (adjusting for age and gender) linear regression models are shown in Table 3. The regressive ASD children had higher scores than non-regressive ASD children in the ABC total score and the scores of sub-items, and the differences in sensory (β = 1.104, 95% CI: 0.120, 2.089, *P* < 0.05), stereotypic behavior (β = 1.388, 95% CI: 0.038, 2.737, *P* < 0.05) and ABC total score (β = 5.122, 95% CI: 0.818, 9.426, *P* < 0.05) were statistically significant.

**TABLE 3:** Differences in autism symptoms in ASD children with and without regression.

Item	R (*n* = 138)	NR (*n* = 889)	β (95%CI)	*P*
**ABC (*n*1 = 134, *n*2 = 847)**				
Sensory	9.00(4.00–12.00)	7.00(3.00–11.00)	1.104(0.120, 2.089)	**0.028**
Relating	16.50(9.00–20.00)	13.00(7.00–18.00)	1.381(−0.114, 2.875)	0.070
Stereotypic behavior	9.50(4.00–14.00)	6.50(2.00–12.00)	1.388(0.038, 2.737)	**0.044**
Language	12.50(5.75–19.00)	11.00(5.75–16.00)	0.916(−0.381, 2.214)	0.166
Social self-help	12.00(9.00–16.00)	11.00(7.00–15.00)	0.807(−0.175, 1.790)	0.107
ABC total score	60.00(39.755–71.25)	48.00(36.00–63.25)	5.122(0.818, 9.426)	**0.020**
**SRS (*n*1 = 117, *n*2 = 759)**				
Social awareness	12.50(9.75–14.25)	12.00(9.00–13.00)	0.289(−0.357, 0.934)	0.381
Social cognition	19.00(16.00–22.00)	18.00(15.00–21.00)	0.784(−0.103, 1.670)	0.083
Social communication	32.50(28.75–42.25)	32.00(26.00–38.00)	1.712(−0.053, 3.477)	0.057
Social motivation	16.50(11.75–20.00)	15.00(12.00–18.00)	0.922(−0.057, 1.902)	0.065
Autistic mannerisms	16.00(10.00–19.25)	12.00(9.00–17.00)	1.297(0.140, 2.453)	**0.028**
SRS total score	97.76 ± 24.81	88.86 ± 22.16	4.991(0.494, 9.487)	**0.030**
**CARS (*n*1 = 120, *n*2 = 739)**				
CARS total score	32.50(30.00–38.00)	33.00(28.00–38.00)	−0.001(−1.317, 1.314)	0.998
**Communication warning behavior total score (*n*1 = 94, *n*2 = 624)**	44.00(30.00–62.50)	40.50(25.00–58.00)	4.553(−0.007, 9.112)	0.050

*Multivariate linear regression was used for adjusted model (adjusting for age and gender); β (95%CI), regression coefficient (95% confidence interval); n1, the sample of regressive group; n2, the sample of non-regressive group. ABC, Autism Behavior Checklist; SRS, The Social Responsiveness Scale; CARS, Child Autism Rating Scale.*

The regressive ASD children also had higher scores in the Social Responsiveness Scale (SRS) total score and the scores of sub-items compared with the non-regressive ASD children, while there were only two statistically significant differences in autistic mannerisms score (β = 1.297, 95% CI: 0.140, 2.453, *P* < 0.05) and SRS total score (β = 4.991, 95% CI: 0.494, 9.487, *P* < 0.05) between the two groups.

The Child Autism Rating Scale (CARS) total score in the regression group was slightly lower than that in the non-regression group, however, the difference is not statistically significant. The regressive ASD children also had a higher score in the communication warning behavior totle score (β = 4.553, 95% CI: −0.007, 9.112, *P* = 0.05).

### Comparison of the Developmental Levels Between Regressive Group and Non-regressive Group

It can be seen from Table 4 that the Revised Children Neuropsychological and Behavior Scale (CNBS-R2016) general quotient (GQ) and DQ of gross motor, fine motor, adaptive behavior, language, and personal-social in the regression group are all lower than the non-regression group. The difference in language was statistically significant between the two groups (β = −5.827, 95% CI: −11.529, −0.125, *P* < 0.05).

**TABLE 4:** Differences in developmental quotient in ASD children with and without regression.

Item	R (*n* = 138)	NR (*n* = 889)	B (95%CI)	*P*
**CNBS-R2016 (*n*1 = 95, *n*2 = 630)**				
Gross motor	73.50(63.75–85.25)	78.00(65.00–89.00)	−2.574(−7.086, 1.938)	0.263
Fine motor	57.00(48.00–66.00)	58.00(47.00–70.00)	−3.708(−7.888, 0.472)	0.082
Adaptive behavior	59.50(45.75–70.25)	62.00(49.00–75.00)	−3.450(−8.141, 1.241)	0.149
Language	44.50(29.75–62.00)	46.00(33.00–67.00)	−5.827(−11.529, −0.125)	**0.045**
Personal society	51.00(40.50–61.00)	52.00(43.00–64.00)	−4.232(−8.728, 0.264)	0.065
GQ	59.50(47.00–66.00)	59.00(50.90–70.25)	−3.965(−8.051, 0.084)	0.055

*Multivariate linear regression was used for adjusted model (adjusting for age and gender); β (95%CI), regression coefficient (95% confidence interval); n1, the sample of regressive group; n2, the sample of non-regressive group. CNBS-R2016,Children Neuropsychological and Behavioral Scale-Revision 2016.*

## Discussion

Although previous studies have gradually paid attention to the regressive ASD, there is still a lack of large sample investigations on its rates and onset of regression, regression subtypes, and disease severity in China. Therefore, this study conducted the first nationwide multi-center study to further describe the onset pattern of regressive ASD and its relationship with the severity of the disease. Our research results showed that although the prevalence rates for regressive ASD was not very high, it was more serious than non-regressive ASD.

Findings from the meta-analysis indicated that regression prevalence varies with different regression classification methods and sampling methods (14). Recent reviews in retrospective studies reported the rate of regression ranging from 10 to 50% (15–17), and overall prevalence rate for regression was 30% (8). However, in some prospective studies, the detection rate of retrogression was as high as 80% (18, 19). Ozonoff et al. analyzed the reason why the results of the two research methods are so different may be that prospective studies can observe earlier and more subtle regressive behaviors (5). Moreover, there were also some prospective studies conducted in high-risk siblings with a family history of ASD, so the incidence of regression was higher (14, 20).

Our research results suggested that the incidence of regression was 13.44%, which was lower than the domestic retrospective research result (32.97%) of Wu et al. (21). The reason may be that our study only included children whose parents had detailed descriptions of specific regression behaviors, and quantified and standardized the language skills of the children before regression; all ASD children who reported language regression before the age of 18 months were excluded, which is more stringent than some studies that use 15 months as the occurring time for language regression (22). According to the definition of language development milestones, generally speaking, “language descriptions” appear in babies at the age of 6–12 months, which is unconscious pronunciation, and there is no clear direction. While the general age of children who have acquired at least 5 different words is 1.5–2 years old. Because parents can easily confuse the above criteria, language regression does not include children whose regression occurring before 1.5 years in this study. In addition, like some other studies (22, 23), our research only focused on the regressions in three aspects: language, social, and motor, and did not include more aspects such as imagination, gestures, fine motor, and adaptability, thus causing a lower regression prevalence.

Regarding the age of onset of regression, Tan et al. reviewed other studies and concluded that the average occurring age was 1.65 years or 19.80 months (8). In this study, parents reported that the average age of retrogression onset was 24.00 months. A large number of retrospective studies are in the form of parents’ reports. Due to recall bias, the age of regression onset reported by parents of older children may be later than the actual age (5, 19). Therefore, more large-scale prospective studies are needed to investigate the regression.

Many studies have focused on the severity of symptoms in children with regressive ASD, but the conclusions are not consistent. Most studies suggested that the core symptoms of ASD in regression children were more severe than the non-regression children (24–26). However, some studies suggested that there was no significant difference in clinical symptoms and disease severity between children with and without regressive ASD (23, 27). The inconsistency of research results may be due to the small sample size and the small age of the included children. The potentially destructive effects of regressive ASD may not be obvious in younger children, and this effect becomes more significant when the children are older and the test scores become more stable.

In our study, the scores of ABC and SRS of the regression group were significantly higher than the non-regression group, indicating that the regression group’s disease was more severe. In addition, we also found that the CNBS DQ scores in the regression group were lower than the non-regression group and the proportion of intellectual developmental impairment is also higher in the regression group, suggesting the regression group ASD children have lower neurodevelopmental levels. Which is consistent with the results of Bradley et al. (28).

Recently, a small-sample prospective study conducted by Martin et al. also found that the regression group and the non-regression group showed different clinical manifestations and prognosis in terms of the severity of autism symptoms and neurodevelopment (29). The regression group in the initial investigation showed more severe ASD symptoms and lower overall developmental levels. After 24 months of follow-up, the differences in overall developmental levels between the two groups persisted. Therefore, it is recommended to use developmental regression as a danger warning signal. Once discovering developmental regression, there is no need to wait for a clear diagnosis, and early intervention should be started immediately. In the future, we should also conduct further research on the pathophysiology and genetic mechanism of the causes of more severe disease and more serious clinical manifestations of regressive autism.

The results of this multi-center study are representative of the developmental regression of children with ASD in our country, and may provide a reference for clinicians and parents. But at the same time, this study also has certain limitations: (1) Because this research is a retrospective study, there is a certain recall bias, and there may be some deviations in the occur age of regression and whether the regression behavior occurred. (2) This study adopted the form of questionnaire survey, which has certain requirements for the comprehension and cognition level of the person who completes the questionnaire, so there may be a certain amount of information bias.

## Conclusion

This multi-center study found that the prevalence of regressive ASD was low, and regressive skills were mainly manifested in language and social. However, due to its more severe clinical symptoms and worse prognosis, both medical staff and parents should actively monitor the development levels of children, so as to identify regression in time, and conduct early intervention training. Next, we will carry out a large-sample prospective cohort study to further explore the more subtle development trajectory of children with ASD, and provide data support for in-depth understanding of the disease pattern of ASD.

## Data Availability Statement

The original contributions presented in the study are included in the article/supplementary material, further inquiries can be directed to the corresponding author.

## Ethics Statement

The studies involving human participants were reviewed and approved by the Ethics Committee of the Children’s Hospital of Chongqing Medical University. Written informed consent to participate in this study was provided by the participants’ legal guardian/next of kin.

## Author Contributions

CH and FY completed the statistical analyses, drafted the initial manuscript, and reviewed and revised the manuscript. TY, JieC, YD, FJ, LW, YH, LL, JZ, XK, MY, QH, JinC, SF, YW, QW, and CJ contributed to the conceptualization and design of the study and supervised the data collection. TL and LC conceptualized and designed the study and reviewed and revised the manuscript. All authors approved the final manuscript as submitted and agreed to be accountable for all aspects of the work.

## Conflict of Interest

The authors declare that the research was conducted in the absence of any commercial or financial relationships that could be construed as a potential conflict of interest.

## Publisher’s Note

All claims expressed in this article are solely those of the authors and do not necessarily represent those of their affiliated organizations, or those of the publisher, the editors and the reviewers. Any product that may be evaluated in this article, or claim that may be made by its manufacturer, is not guaranteed or endorsed by the publisher.
